# Managing non-communicable diseases at health district level in Cambodia: a systems analysis and suggestions for improvement

**DOI:** 10.1186/s12913-016-1286-9

**Published:** 2016-01-27

**Authors:** Bart Jacobs, Peter Hill, Maryam Bigdeli, Cheanrithy Men

**Affiliations:** 1 Social Health Protection Programme, Deutsche Gesellschaft für Internationale Zusammenarbeit (GIZ), c/o NIPH, No.2, Street 289, Khan Toul Kork, P.O. Box 1238, Phnom Penh, Cambodia; 2 School of Population Health, The University of Queensland, Herston Road, Herston, 4006 Brisbane, Australia; 3 Alliance for Health Policy and Systems Research (HSR/HIS), World Health Organization, 20 Avenue Appia CH-1211, Geneva 27, Switzerland; 4 Chean & Jaco Consulting Ltd, Street, #457 Group 1, Thnout Chrum Village, Sangkat Beung Tumpoun, Khan Meanchey, Phnom Penh, Cambodia

**Keywords:** Non-communicable diseases, District health system, Access, Medicine, Diagonal approach

## Abstract

**Background:**

Cambodia developed its public health system along the principles of the district model and geared its services towards managing communicable diseases and maternal and child health issues. In line with other countries in the region, non-communicable diseases have emerged as a leading cause of adult mortality. We assessed the current capacity of the Cambodian district health system to manage hypertension and diabetes, with a focus on access to medicine for these chronic conditions.

**Methods:**

A case study whereby in three purposely selected districts in an equal number of provinces a total of 74 informants were interviewed: 27 health care providers and administrators, 30 community representatives and 17 managers of specific non-communicable diseases interventions and social health protection schemes. Questions related to the World Health Organization’s health system building blocks. Data analysis involved coding, indexing, charting and mapping the data. Following these exercises all information was analysed by kind of respondent and their respective answer to the question concerned. Responses by respondents of three groups of interviewees were compared when appropriate. At 14 health centres and 3 district hospitals the availability of key medicines for hypertension and diabetes in accordance with the National Essential Drug List was assessed. This was also done for essential tools and equipment to diagnose these two conditions.

**Results:**

Although there was agreement amongst nearly all interviewees that non-communicable diseases were prevalent, the district health system, including all health systems building blocks and the referral system, was inadequately developed to effectively deal with these conditions. Medicines supply was erratic and the quantity provided allowed for few patients to be treated, for a short period only, mainly at secondary or tertiary level.

**Conclusions:**

Because of the public health, social and economic importance of non-communicable diseases, a rapid response is required. Given the current Cambodian situation, such response may initially be a diagonal approach, with non-communicable diseases services integrated in the National HIV/AIDS Programme. This should happen together with a reorientation of the health system to enable a horizontal approach to non-communicable diseases management in the long term.

## Background

Chronic non-communicable diseases (NCD) pose a major public health problem in Southeast Asia. In 2008 NCD were estimated to account for 61 % of total disability-adjusted life years of people in the economically active age group of 15–59 years and 84 % for those aged older [1]. The impact of NCD in low- and middle-income countries (LMIC) is of such proportions that it is estimated to account for 80 % of global deaths [2]. The epidemic(s) of NCD presents significant challenges to health systems geared towards dealing with communicable diseases and reproductive and child health issues [3]. Unlike these conditions, NCD management requires patients to have prolonged and regular contact with the health system, and because of their chronicity, lifelong medication therapy is required [4]. With health systems in LMIC oriented towards acute, reactive care, insufficient attention has been paid to health promotion and few comprehensive policies for NCD have been developed or implemented [5].

For patients and their families, repeated visits to health providers for management of NCD create a considerable economic burden. If LMIC health systems are to effectively engage what is the unrecognized bulk of their burden of disease, they will need to reorient to accommodate the needs of NCD patients: drug management for current conditions, screening and controlling for complications and co-morbidities, and actively promoting an appropriate life style, with a view to complementing drug management and secondary prevention of complications [3, 4]. The most effective mechanism to lower the projected workload and financial burden on the health system, while enabling NCD patients affordable access to continuous care, is through primary health care: continuing integrated care in the community [1, 6]. The current weak health systems in many LMIC, not yet reoriented towards meeting the NCD challenge, have been unable to harness effective NCD management through their primary care systems [7, 8]. Not surprisingly, Vialle-Valentin and colleagues [9] found that less than half of people with NCD in five LMIC had access to medicines for their condition.

In this paper we examine the capacity of the Cambodian district health system to deal with diabetes and hypertension, two common conditions amongst rural and urban adults [10] with prevalence estimates of 5–11 % and 12–25 % respectively. The district health system constitutes the model for delivery of public health services to the Cambodian population. In rural areas, home to 80 % of the total population, the district health system is considered the major vehicle for primary health care. We focus in particular on access to medicines within the district health system for these concerned conditions as such access is considered crucial for the system’s ability to manage NCDs and thus reduce their public health and socioeconomic impact.

### Cambodia’s district health system

Cambodia’s public health system is three-tiered, based on the district health system model and consisting of the central ministry, provincial health offices and operational health districts (OHDs). The district health model was reinforced in 1995 when the Ministry of Health launched its Health Coverage Plan, which divided the country into operational health districts as the cornerstones for public health service delivery. Each OHD is intended to cover a population of 100,000 to 200,000 people and may cut across administrative district boundaries. The OHD consists of health centres backed by a referral hospital. The Health Coverage Plan was soon accompanied by Guidelines for Developing Operational Districts [11], which specified the roles and terms of reference for all actors within the OHD, including administration by the Director and two Vice-Directors, the District Health Technical Advisory Committee and health facilities. Provincial Health Offices are responsible for the equitable distribution and effective use of available resources; and the provision of technical support for the development of OHDs.

A health centre covers 5000 to 20,000 people belonging to one or more communes and provides a Minimum Package of Activities (MPA). These activities are mainly preventive and basic curative services with an emphasis on maternal, newborn, child and reproductive health supplemented by outreach services and specific activities for vertical programs. The curative services include consultations and primary diagnosis, emergency first aid, chronic disease care for HIV and TB. In 2013, 1049 health centres provided the full range of MPA services. Referral Hospitals are established within each OHD, with one provincial referral hospital in the provincial capital. The hospitals are classified into three levels according to their package of medical activities.

The Guidelines for Developing Operational Districts [11] and the Charter on Health Financing [12] endorsed community participation in the management of health centres. Co-management of health centres was to be achieved through community representation and participation in the planning, implementation and use of health services by means of the Health Centre Management Committees (HCMCs) and Village Health Support Groups (VHSGs). The HCMC’s main roles and responsibilities are to ensure provision of preventive and curative care; to improve quality of care and its utilization; and to create a transparent accountability system for efficient use of health centre resources, including user fees. Village Health Support Groups should exchange information between the health centre and the community and conduct health education.

User fees are charged by nearly all public health facilities but are nominal in nature and mainly intended as a staff incentive as the government subsidizes the running expenses, including medicine. Essential drugs are procured by the Ministry of Health and provided to public health facilities through the MOH Central Medical Stores. Medicines are to be provided for free to patients at public health facilities.

In respect to NCD, the second Health Strategic Plan 2008–15 [13] mentions as one of its three goals the reduction of the burden of NCD. This is to be achieved, amongst other strategies, by strengthening capacity to provide integrated NCD care and secondary prevention at primary care level. The 2013–20 Strategic Plan for Prevention and Control of NCD [14] refers to prior accomplishments for NCD control as the establishment of chronic disease outpatient clinics (CDC) in 8 provincial hospitals, initiation of peer educator networks for diabetes and hypertension in 10 OHD, development of a training curriculum for health professionals working in diabetes clinics and revision of the MPA to allow health centre staff to treat patients with mild hypertension. The WHO Package of Essential NCD Interventions is to be employed as the basic package for NCD education, screening and treatment. This package is to be piloted in health facilities from 2013 onwards, with a view to progressively introducing it to all health facilities from 2015 onwards [14].

The peer education network was introduced by a non-governmental organization (NGO) in 2007 and consists of networks of peer educators in 13 OHD. By use of this approach the NGO facilitates the recruitment and training of patients into peer-educators and has instigated systems to supervise and monitor them. In tandem it facilitates affordable access to public medical services through mechanisms that allow outpatient consultations, laboratory services and a continued affordable supply of medicines by use of a revolving drug fund [15]. The CDCs are outpatient clinics at tertiary public referral hospital level –with the exception of two district hospitals- that provide integrated, patient-centered care for people with diabetes and/or hypertension [16]. The WHO Package of Essential NCD Interventions in the Cambodian pilot mentioned above was to focus on reducing risk factors for NCD and clinical management of hypertension and diabetes type 2. However, by the end of 2014, the WHO Package of Essential NCD Interventions had yet to be introduced in Cambodia.

## Methods

This study is part of a wider research that sought to identify realistic strategies to enable the Cambodian rural population affordable access to NCD medicines by considering existing risk protection schemes, health system configurations and socioeconomic and cultural characteristics. Here we report on the assessment that sought to identify district health system components that can be built upon or improved to ensure durable and affordable access to medicine for patients with NCD. These include interactions between subcomponents of building blocks: service delivery, financing, governance, health workforce, health information system, and supply management [17].

The study took place in three purposively selected health districts in three provinces. Selection criteria were rural location and the presence of social health protection schemes for the informal sector population—Health Equity Funds for the poor and voluntary insurance schemes for the non-poor. Health Equity Funds are third party arrangements that reimburse public health providers the user fees of services rendered to eligible poor beneficiaries. Two districts were selected because they had interventions specifically designed to enable patients with NCD access to treatment: peer-education network and CDC. Table 1 displays the districts selected, associated schemes, NCD interventions and population data.

**Table 1:** Study sites’ specifications

Health District	Population	Social Health Protection Scheme (% population covered by scheme)	Chronic Disease Intervention
Kirivong	224,401	VI (3.6), HEF (20.6)	CDC, PEN
Samrong	209,999	VI (15.9), HEF (28.8)	-
Baray-Santuk	253,260	HEF (24)	CDC, PEN

*CDC* chronic disease clinic, *HEF* health equity fund, *PEN* peer education network, *VI* voluntary insurance

A combination of key informant interviews and facility assessment was applied to examine these district health systems’ ability to ensure access to treatment for NCD patients.

### Key informant interviews

A variety of informants, totalling 74, categorised along three groups were interviewed in September 2013 by trained experienced interviewers. Interviews took place at the interviewees’ health facility or administrative offices. These groups and respective interviewees were (Table 2):

**Table 2:** Kind and number of interviewees per category and health district

	Baray-Santuk	Kirivong	Samroang	Total
Provincial Health Department	1	1	1	3
Operational Health District	1	1	1	3
Provincial Hospital	1	1	1	3
District Hospital	1	1	1	3
Health Centre	5	5	5	15
*Subtotal*	*9*	*9*	*9*	*27*
Village Health Support Group	5	5	5	15
Health Centre Management Committee	5	5	5	15
*Subtotal*	*10*	*10*	*10*	*30*
Peer Education Network	3	3	0	6
Chronic Disease Clinic	2	1	0	3
Voluntary Insurance Scheme	0	2	2	4
Health Equity Funds	2	0	2	4
*Subtotal*	*7*	*6*	*4*	*17*
Total	26	25	23	74

- Health care providers and administrators (*n* = 27). The directors of each Provincial Health Department, Operational Health District, Provincial Hospital and District Hospital (total *n* = 12 directors). At each Operational Health District five health centres were randomly selected and at each facility the respective chief was interviewed.
- Representatives of the Village Health Support Groups (VHSG) and chairmen of the Health Centre Management Committees (HCMC; *n* = 30). For each of the 15 selected health centres, representatives of the respective community participation structures were interviewed.
- Scheme managers (*n* = 17): managers or staff members of the social health protection schemes and the NCD interventions.

Indicators for examining the building block’s contribution to the district health systems’ ability—or inability—to cater for the needs of people with NCD were derived from the Maximizing Positive Synergies Collaborative Group [18]. The indicators were synthesised from research undertaken by this collaborative group, comprising representatives of multilateral, bilateral, research and implementation institutes based in about 28 countries of various stages of socioeconomic development. These indicators are displayed by building block in Table 3.

**Table 3:** Indicators of health systems building blocks

*Service delivery*:	access, equity, quality
*Financing*:	domestic budget allocations, out-of-pocket expenditure
	aid effectiveness, amount of funding
*Governance*:	planning and coordination, community involvement
*Health workforce*:	production and strengthening, distribution
*Health information system*:	availability and accuracy, use and demand, innovation
*Supply management systems*:	procurement and distribution, quality

The questionnaires were structured with open-ended and pre-coded questions. Questions for interview were structured along the indicators specified in Table 3. The questions are summarised in Table 4. Analysis was undertaken manually by adopting the indicators of the building blocks as a priori codes (Table 3). Responses to open-ended questions were coded during analysis and thereafter treated as quantitative information. The analysis involved coding, indexing, charting and mapping the data. Following these exercises, all information was entered in MS Excel and analysed by kind of respondent and their respective answer to the question concerned. Interviewees were stratified for analysis as service providers, community representatives and scheme managers. For the latter, results were analysed by specific scheme, as well as aggregated. Results for community representatives and scheme managers are provided were relevant; that is, when they were deemed to have knowledge regarding the question.

**Table 4:** Overview of questions

Service delivery
• Tools and equipment available to diagnose hypertension and diabetes
• Ability to manage patients with NCD
• Population awareness of NCD services at public facilities
• Stage of disease development when patients are diagnosed with NCD
• Management of NCD complications
• Special services for patients with NCD
• Major constraints for adequately managing patients with NCD in public sector
Financing
• User fee charges per service for NCD
• Annual budget per level of health administration and health facility for NCD
• Presence of donor-supported programs for NCD
• Opinion about effectiveness of social health protection schemes for enabling access to medicine for NCD and financial protection
Governance
• Presence of national policy or strategy for the treatment and long-term care of NCD
• Availability of treatment guidelines for management of NCD
• Provision for NCD in planning cycle
• Provision of NCD in budgeting
• Community involvement in management of NCD
• Community initiatives for mobilising resources for patients with NCD
• Promotion/education campaigns/activities on NCD
Workforce
• Presence of staff members who received training in management of NCD
• Whether a staff member is in charge for NCD in the facility or administration
• Educational background of this person
• Additional responsibilities beside NCD
• Specific training on NCD
• Refresher courses for NCD
Health information system
• Specific mention of NCD in the health management information system
• Presence database system to monitor patients’ adherence with treatment
• Availability recall system for dropouts
Supply management
• Provision of medicines for managing NCD by Central Medical Stores (CMS)
• Supply of medicine for NCD in accordance with requirements (needs)
• Time period for which a patient receives treatment for NCD at each outpatient consultation
• Number of patients to be treated with one quarterly supply by CMS
• How long the supply last on average
• Where the facility refers patients when NCD drugs are not available
• Supply of consumables for diagnosing and monitoring patients with NCD by CMS

### Facility assessment

To assess the availability of medicine, we used WHO’s instrument for assessing, monitoring and evaluating country pharmaceutical situations [19] with its focus on 2 variables: availability of key medicines for diabetes and hypertension, and their stock out. This assessment was conducted at each of the selected health facilities (14 health centres and 3 hospitals) —with the exception of one health centre in Samrong health district that was closed when visited. An experienced medical doctor visited the facilities to collect the data.

Assessment of the availability of hypertension and diabetes medicines was based on the list of essential medicines for hypertension and diabetes in MPA & CPA facilities. The five main groups of antihypertensive medicines were considered: Thiazide-type diuretic (Hydrochlorothiazide), calcium channel blocker (CCB, Amlodipine and Nifedipine), angiotensin-converting enzyme inhibitor (ACE inhibitor, Captopril and Enalapril), Beta blockers (Atenolol), and angiotensin receptor blocker (ARB, Losartan). For antidiabetic medicine, three groups were considered: insulin sensitizer (Metformine), insulin secretagogues (Glibenclamide) and insulin injection (rapid/intermediate acting).

The tools and equipment required to diagnose the two conditions concerned were derived from the WHO document Package of Essential Non-Communicable Disease Interventions for Primary Health Care in Low-Resource Settings [20].

### Ethics

Ethical approval was obtained from the Cambodian National Ethics Committee for Health Research (0008 NECHR) and the Research Ethics Review Committee of the World Health Organization (RPC 551). Written informed consent was obtained from all study participants.

## Results

The results describe our evaluation of district health system components that are necessary for the sustainable and affordable access to medicine for NCD patients, by health system building block.

### Service delivery

Chronic non-communicable diseases were recognized as prevalent by 71 % of scheme managers and 78 % of providers, but 26 % and 35 % respectively were of the opinion that such patients presented themselves only when the disease was already well developed. This contrasted with the 60 % of community representatives who considered late presentation common. The providers’ opinion contrasted again with that of the community representatives when queried concerning their ability to manage NCD: 37 % of providers rated their NCD management ability as excellent and 52 % mediocre, while none of the community representatives considered NCD management to be excellent, and 60 % found it to be inadequate.

The audit of the availability of essential technology for diagnosing and managing NCD corroborated the community representatives’ concerns. All facilities had a sphygmomanometer; all hospitals—but none of the health centres—were able to measure blood glucose levels, or had urine tests for protein; but only the provincial hospitals were able to measure haemoglobin A1C. All facilities had the necessary equipment to collect the information for calculating body mass index.

In interviews with providers and scheme managers concerning services for people with NCD (Table 5), it became apparent that the clinical aspects of NCD-management- screening, diagnosis, treatment, and follow up- constituted the main focus of services. With screening, 81 % of providers claimed it was available compared to 59 % of scheme managers; 93 % of providers confirmed referral of cases, compared to 47 % of scheme managers. Providers tended to report limited management of complicated cases, inadequate counselling of patients, and insufficient financial incentives to enable patients’ continuing access to affordable medication. The limited availability of medicines for NCD was the most mentioned constraint, and a third mentioned challenges with diagnostic tests and following-up patients. Primary prevention was reported by only one scheme operator—and no other informants.

**Table 5:** Service provision for NCD patients and perceived challenges to adequately manage them: perspectives from providers and scheme managers

	Providers		Scheme managers	
	*N* = 27		*N* = 17	
	N	%	N	%
What services do you provide for NCD patients?				
Screening	22	81	10	59
Diagnostic	16	59	9	53
Treatment	18	67	10	59
Follow-up	16	59	9	53
Management of more complicated cases	8	30	2	12
Referral of complications	25	93	8	47
Incentives to stay within the program,	2	7	2	12
Counselling, lifestyle changes, peer support	8	30	9	53
Primary prevention of diabetes and hypertension	0	0	1	6
What are the challenges to adequately manage NCD patients?				
Medicine availability	18	67	15	88
Diagnostic tests/screening	10	37	7	41
Follow-up	9	33	6	35
Management of more complicated cases	7	26	2	12
Referral of complications	4	15	5	29
Incentives to stay within the program	1	4	2	12
Counselling, lifestyle changes, peer support	6	22	6	35
Population awareness	8	30	5	29
Late diagnosis (advances stage)	4	15	2	12
Establishment of permanent health facility for patients with CNCD	0	0	1	6
Need specialized medical staff to treat patients with diabetes	7	26	3	18
Sufficient medical devices	4	15	3	18

### Financing

Few of the facilities examined had an annual government budget for NCD, and those budgets available were minimal (Table 6). One provincial health department had a budget of US$1600; its respective provincial hospital US$2400; and the district hospital US$1800. Only 2 health centres reported an annual budget of US$2000, but none of the operational health district administrations had any.

**Table 6:** Fees charged by public health facilities and NCD interventions (in Khmer riels)

Type of Health Facility	N^a^	Mean	SD	Median	Minimum	Maximum
Consultation Fees						
Provincial Hospital	3	29,500	43,769	6000	2500	80,000
District hospital	3	17,500	28,340	3750	2500	60,000
Health center	13	1885	893	2000	500	3000
Peer education network	2	4375	1750	3500	3500	7000
Chronic Disease Clinic^b^	2	2667	289	2500	2500	3000
Diagnostic Fee						
Provincial Hospital	2	6250	5303	6250	2500	10,000
District hospital	1	10,000	0	10,000	10,000	10,000
Peer education network	2	2720	2418	2000	1300	7000
Medicine fee						
Provincial Hospital	1	2500	0	2500	2500	2500
District hospitals	0	0	0	0	0	0
Health center	1	2000	0	2000	2000	2000
Peer education network	2	8860	5104	10,000	1300	15,000
Laboratory tests						
Provincial Hospital	2	3250	1061	3250	2500	4000
District hospital	2	6500	2121	6500	5000	8000
Health center	1	1500	0	1500	1500	1500
Peer education network	2	22,100	224	22,000	22,000	22,500

^a^denotes the number of facilities/schemes charging fees. For peer education networks and chronic disease clinics averages are given based on the sum of figures provided by the respective respondents; ^b^consultation fee covers medicines and laboratory tests

With the exception of two health centres, all facilities charged consultation fees from patients for NCD-related services, ranging from KHR2000 (US$0.5) to KHR6,000 (US$1.5) (Table 6). Diagnostic fees only were charged by five health facilities, while one health centre and one provincial hospital insisted patients pay for NCD medicines, contrary to the national guidelines. Two thirds of hospitals levied fees for laboratory tests, ranging from KHR3,250 (US$0.8) to KHR6,500 (US$1.65).

Interviewees reported that no external support was provided for NCDs, with the exception of the peer education network, mentioned in the provinces and districts were it was operational.

All scheme managers considered that the social health protection schemes were effective in enabling access to care for people with NCDs, with 81 % of providers agreeing. This positive perception was mainly ascribed to the health equity funds and the fact that they enable free access to treatment—though only 73 % of the community representatives thought the schemes effective, and two thirds of respondents or less were confident they provided free access.

### Governance

Three quarters of providers, but only 41 % of scheme managers were aware of any provision for NCD in the annual planning cycle for operational health districts and respective facilities. None of the community representatives were aware of this. Only 29 % of managers and 15 % of providers mentioned that there was a provision for NCDs in the budgeting; and no community representatives were aware of this provision.

Around half the providers and managers reported that the community was involved in management of NCD, though only one third of community representatives agreed. There was, however, relative concurrence that there were no special initiatives to mobilise human or financial resources from the community, with only 10 % of community representatives, 12 % of managers and 15 % of providers reporting any such initiatives.

### Workforce

All providers, and most health centre chiefs (80 %) were confident that NCDs were mentioned in the MPA and CPA guidelines, but in one Provincial health department and its respective facilities and district administration, nobody had received formal training in management of NCD. Overall, nearly half of health centres (47 %) did not have anybody employed who had completed formal NCD management training. The situation was especially worrying for the social health protection schemes, as none of the voluntary insurance or health equity fund managers had any formal training on NCDs. Of the 15 providers who received training in management of NCDs, 67 % were medical doctors and the remainder nurses. The Ministry of Health provided all training, but 74 % of the providers mentioned that there had been no refresher courses organised.

Interviews with the providers to identify staff members responsible for NCD presented a diverse picture:

- *Provincial Health Department*s (PHD): at two departments, a deputy director was in charge and at one a technical officer. Both were medical doctors.
- *Operational Health District*: at one district the hospital director was in charge, one had a technical officer and one considered their respective PHD director responsible. All were medical doctors.
- *Provincial hospital:* one hospital reportedly had nobody for NCDs, one considered their respective PHD director responsible and another, the hospital director. All were medical doctors.
- *District hospital*: one had the hospital director in charge for NCDs, one a technical officer, all of whom were medical doctors, and one had nobody responsible for NCDs
- *Health centres*: a third of the health centres (*n* = 5) had nobody responsible for NCDs. In others, the health centre chief was responsible (*n* = 3), a staff member (*n* = 3), the hospital director (*n* = 1) or hospital technical officer (*n* = 1), or the district director (*n* = 1), or the deputy (*n* = 1). Only 3 (out of 10) were medical doctors.

### Health information system

Fifty nine percent of providers considered that NCDs are reported in the health information system (HIS), but a further third indicated that these conditions were included with many other non-specified conditions. Two thirds of managers -including all CDC and peer-education network managers- were aware that the HIS reported NCDs. Community representatives in general (77 %) were not aware that NCDs had been included in the health information system.

Few facilities reported a patient monitoring system in place: only one district hospital, one provincial hospital and four health centres. CDC and peer-educators were all compliant, but not voluntary insurance and HEF managers. Again, all of the peer education network managers had a recall system for treatment dropouts, though only one CDC operator, and a third of secondary and tertiary level providers mentioned the availability of a recall system.

Interviewees were queried whether the drug supplies matched demands; that is, whether the supply accorded with the number of patients. On average, about half of providers mentioned this was the case: 73 % of health centre chiefs but none of the district hospitals. Only the peer-education network managers reported a supply sufficient to match their demands while other scheme managers could not confirm this.

### Supply management system

All providers reported receiving medicines from the Central Medical Stores for managing NCDs. The figure for scheme managers was 65 %, though only 25 % of voluntary insurance and HEF representatives were certain that Central Medical Stores provided these drugs. Community representatives were not aware: only 10 % could confirm this, with the remainder having no idea.

Health centres reported that their available supplies would offer a median of only 3 days of medicine to NCD patients. The hospital representatives mentioned that they were able to provide drugs to treat 5 to 7 diabetes patients per quarter only. These figures contrast sharply with those of the NCD intervention schemes, which indicated that they were able to provide 30-day supplies to all patients (Table 7).

**Table 7:** Reported amount of medicines supplied to patients and estimated number of patient who can be managed per quarterly supply by Central Medical Stores

	Mean (SD)	Median (Min-Max)
Amount of medicine supplied to patients for each visit (in days)		
Provincial hospital	19 (12)	20 (7–30)
District hospital	9 (6)	7 (4–15)
Health centre	4 (1)	3 (3–5)
Peer education	28 (6)	30 (15–30)
Chronic disease clinic	27 (6)	30 (20–30)
Number of patients who can be treated with each supply by CMS: diabetes		
Provincial hospital	5 (0)	5 (5–5)
District hospital	6 (1)	7 (5–7)
Peer education	859 (1757)	80 (5–4000)
Chronic disease clinic	578 (85)	600 (485–650)
Number of patients who can be treated with each supply by CMS: Hypertension		
Provincial hospital	6 (0)	6 (6–6)
District hospital	118 (182)	16 (11–328)
Health centre	16 (22)	10 (3–90)
Peer education	569 (1080)	100 (7–2500)
Chronic disease clinic	309 (117)	350 (177–400)

*CMS* central medical stores, *SD* standard deviation

Drug availability was an issue at health facilities (Table 8) with none having all required essential drugs available, and the chronic disease clinics also experienced stock-outs. Reported availability of drugs in referral hospitals and CDC were largely confirmed by observation. The peer education network, through its revolving drug fund, performed best concerning the availability of essential drugs for NCD.

**Table 8:** Reported and observed available essential medicine for hypertension and diabetes

Medicine	Health centre		District hospital		PH	CDC	PEN
	Interview^a^	Assessed^b^	Interview	Assessed			
	N (%)	N (%)	N (%)	N (%)	N (%)	N (%)	N (%)
*Hypertension*							
Hydrochlorothiazide	5 (33)	12 (86)	3 (100)	1 (33)	0	1 (50)	2 (100)
Amlodipine	1 (7)	1 (7)	2 (67)	2 (67)	1 (33)	2 (100)	2 (100)
Atenolol	1 (7)	2 (14)	3 (100)	3 (100)	2 (67)	2 (100)	2 (100)
Captopril	NEDL		0	2 (67)	1 (33)	1 (50)	2 (100)
Enalapril	NEDL		1 (33)	3 (100)	1 (33)	2 (100)	2 (100)
Losartan	NEDL		0	0	0	0	1 (50)
Nifedipine	NEDL		1 (33)	2 (67)	0	0	0
*Diabetes*							
Metformine	NEDL		1 (33)	2 (67)	2 (67)	2 (100)	2 (100)
Glibenclamide	NEDL		1 (33)	2 (67)	1 (33)	1 (50)	2 (100)
Insuline NPH	NEDL		0	1 (33)	1 (33)	2 (100)	2 (100)

*CDC* chronic disease clinic, *PEN* peer education network, *PH* provincial hospital, *NEDL* not on essential drug list; ^a^15 facilities; ^b^14 facilities

At the health facilities, stocks reportedly lasted 1 month on average; chronic disease clinics had stocks that would last 2 months. When they had no medicine available, 25 (93 %) provider respondents mentioned referring the patient to a higher-level facility. This was also the case for tertiary facility providers. The remainder would propose the patients buy drugs on the market. 75 % of social health protection scheme managers would also refer the persons to higher-level providers.

In the case of stock out, only 41 % (11) of providers would resort to special measures to ensure availability of medicine, including writing a letter to health administrators (*n* = 3) or using the revenue from user fees to purchase additional medicine (5). Only one social health protection scheme operator reportedly would take such action.

## Discussion

Goyet et al. [21] conducted a one and a half year prospective study in a rural Cambodian setting in 2010 and found that NCDs were responsible for 52 % of adult deaths. Despite their findings, our study indicates that, similar to Bangladesh, Vietnam, Tanzania, India, Indonesia [7, 22–25], there is hardly provision at primary level to manage NCD.

Although the focus of this study was on access to medicine for NCDs, the requirement to access comprehensive treatment -including counselling and monitoring of the patients’ status -should not be overlooked [5]. Provision of such comprehensive treatment is challenging in absence of adequate financial resources. It is therefore worrisome that only four health facilities (2/6 hospitals, 2/15 health centres) reported an annual budget for NCD management. The annual amounts available per facility equalled about US$2,000 or US$167 per month. All hospitals charged fees for consultations at about US$1–1.5 though the maximums mentioned (US$15–20) suggest that these amounts may vary considerably. Only one Provincial Health Department but none of the OHD administrations mentioned the availability of a budget for NCD, an issue that is not conducive for good management practices.

Few providers were trained in managing NCDs. No refresher courses were available; a third of the hospitals and health centres had nobody in charge for NCD while the description of responsibilities at provincial and district health administrations suggested suboptimal commitment. This situation is not dissimilar from that observed in a health district in neighbouring Vietnam [23]. Only 6 (of 21) health facilities reportedly had a patient monitoring system in place while only two hospitals had a recall system for patient dropouts. Social health protection schemes were not involved with such activities. Monitoring to ensure the sufficiency of NCD medicine supplies was poorly handled.

Despite the lack of certainty of providers, the Central Medical Stores do provide medicine for NCDs. If the estimates provided by hospitals are accurate, and they are only able to manage 5 to 7 patients per quarter, with stock lasting one month on average, clearly the levels of supply of NCD medications is inadequate. None of the health facilities had all essential drugs available. Health centres could only provide medicine for hypertension, for an average of 10 persons, though gave only drugs for 3 days. In case of non-availability of drugs at health facilities, patients would be referred to higher level, although only a third of provincial hospitals reported having the required medicine, also for 7 persons on average. Patients responding to such a referral run a high probability of their needs not being met. Few providers could get sufficient medicine within the public sector by other administrative routes, while the social health protection scheme managers were reluctant to get involved. As such it is highly likely that patients meet the deficits for medicines in the private sector, or go without.

A partnership between the health system and communities has been advocated [3, 4] to reduce the economic burden of managing NCDs through continued care within communities as well as to promote treatment retention. The CDC do not have an effective link with communities, which may be why 34 % of diabetes patients were lost to follow over a 3-month period [16] and 42 % of hypertension patients over a 24 month period [26]. For the Peer Education Network, for which community engagement is an integral part of the approach to treatment of NCD, 97 % of participants reported compliance with medication requirements [15]. Community engagement should clearly be strengthened, however, only a third of the representatives mentioned being involved in NCD-related activities and the precise nature of this involvement was not ascertained. Only 10 % of community representatives mentioned mobilising resources for NCD.

We assessed three operational health districts in three provinces, purposively selected because of the presence of social health protection schemes for the informal sector and/or NCD-interventions. Despite these criteria, provisions for NCD treatment were minimal. The study raises real concerns as to how the Cambodian public health system could enable access to appropriate treatment for people with NCD in the near future. Opening additional CDC at provincial hospitals is unlikely to improve the situation due to travel and opportunistic costs involved, making it costly for patients, and resulting in high attrition rates. Non-delegation of a set of treatment activities to primary level imposes costs on the health system while complications arising from insufficient treatment of NCDs will be dear for society. Services might be brought closer to patients through a public-private partnership [27], as private providers constitute the main source of treatment in Cambodia [28]. For such an approach to be effective, a strong regulatory framework for the private health sector has to be developed and enforced, and operating together with an accreditation system. To date, neither is yet in place.

A diagonal approach could be an appropriate strategy to timely correct the situation. Such approach implies using vertical interventions while concurrently strengthening the health system [29–31]. Related to the diagonal approach is the concept of integrating services to optimise synergies, especially for managing more demanding chronic disorders such as NCDs and HIV/AIDS [29]. Such integrated approach between HIV/AIDS programmes and NCD treatment is increasingly advocated, sometimes with reference to the Cambodian integrated approach in CDCs [6, 24, 32, 33].

The rationale behind integrating NCD management in HIV/AIDS control programs in low-and middle income countries rests on the fact that the rolling out of antiretroviral therapy for people living with HIV has transformed the lethal condition into a chronic non-fatal and essentially manageable disease [24]. Treatment and care for people living with HIV has been decentralized to primary health facilities. The success of the rapid expansion of antiretroviral therapy in challenging environments has been ascribed to pragmatic approaches, including simplification and emphasis on core functions, standardization, decentralization, task sharing, minimization of laboratory requirements, and monitoring patients and outcomes [32]. As such, the required infrastructure for providing lifelong treatment has been established [33].

Cambodia does have an excellent HIV/AIDS control program. It is operational at all health centres and public hospitals in the country and has extensive links with the communities through home-based care networks, peer support groups and NGOs, all of which are actively involved in the control program [34]. The program established a ‘linked response’ that has been rolled-out nationwide with the objective of promoting management of sexually transmitted infections amongst pregnant women, voluntary testing and counselling for HIV to prevent mother-to-child transmission and strengthening reproductive health services [35]. Challenges encountered with the HIV/AIDS control program include heavy reliance on external funding –up to 87 % in 2012- and issues with logistics and supply management of HIV medicines.

Given its public health importance, integrating NCD interventions into the HIV/AIDS control program could be the speediest way to address the issue of insufficient NCD services at health district level. While employing such a strategy as a bridge towards a whole systems approach for NCD treatment, one should consider working towards the model offered by the peer education network, its sustainability evidenced by its treatment compliance rates. Similar to the peer education network, involvement of communities should be considered early on, to enable continued care [4]. Task shifting can greatly aid to allow for continued care, which reduces the burden to health workers and improves compliance [36]. Meanwhile all elements within the building blocks will have to be strengthened to allow the health system effectively manage NCDs. Sufficient funds have to be allocated, through rearrangements of the existing budget and by attracting donor funding. A provision for NCD will have to be included in the budgeting cycle.

One possibility to attract external funding could be through international co-financing arrangements, similar to the Global Fund to fight AIDS, Tuberculosis and Malaria [37, 38]. For this to happen concerted action is required through advocacy, establishing effective partnerships and fostering political support [39], to ensure that NCDs receive proportionate resourcing as one of 9 targets under the proposed Sustainable Development Goal (SDG) for health: the reduction by one third of premature mortality from non-communicable diseases by 2030 [40]. Concerns that NCDs might receive insufficient attention -and thus funding- if absorbed under a broad umbrella goal of universal health coverage or access [41, 42] now appear less likely, though NCDs will continue to compete for resources in this expanded health goal and an extensive SDG agenda.

Once NCD services are available at district level the existing social health protection schemes for the informal sector population—voluntary insurance and health equity funds—should be reconfigured to effectively provide financial protection to patients with NCDs. Currently these schemes do not offer much financial protection for their beneficiaries with NCDs as no special activities are done to cater for their needs. Special activities may include expansion of the health equity fund coverage to people with NCD, reimbursement of transport costs to health facilities, monitoring adherence to treatment and ensuring availability of essential NCD medicine.

Our study took place in three of the country’s 81 OHDs only whereby the findings may not be nationally representable and caution should be exerted to generalize them. However, administration and allocation of government resources are largely centralized at national level, with limited decision-making discretion at lower levels [43], whereby dissimilarities with other OHDs are unlikely. Contrary, the districts are advantaged in terms of support for social health protection, and in two cases, have specific NCD interventions, so they represent contexts in which we would expect NCD management to be optimized.

## Conclusion

At this point, the Cambodian district health system is unable to manage NCD, despite their increasing prominence, accounting for more than half of adult deaths. Apart from the peer education network initiative, the community has not been engaged. Given the public health, social and economic importance of NCD, timely action is required to appropriately manage these conditions and to enable affordable access to related medicines. In light of the current situation, a diagonal approach, whereby NCD management is initially integrated in the HIV/AIDS control program, could be considered. While addressing access to comprehensive NCD treatment through this integrated, though vertical approach, the health system should be reoriented to effectively deal with preventive, promotive and curative aspects of NCDs. In the long run this should allow for a horizontal integrated approach at primary level, possibly in collaboration with a well-regulated private sector. Reconsidered social health protection scheme arrangements could also assist in ensuring affordable access to treatment of NCDs.

## Abbreviations

CDC: chronic disease clinic
CMS: central medical stores
CPA: complementary package of activities
HEF: health equity funds
HCMC: Health Centre Management Committee
LMIC: lower- and middle-income countries
MOH: Ministry of Health
MPA: Minimum Package of Activities
NCD: Non-Communicable Disease
NGO: non-governmental organization
OHD: operational health district
PEN: peer education network
PHD: Provincial Health Department
SDG: sustainable development goals
VI: voluntary insurance.
